# Treatment-Resistant Wide-Complex Tachycardia in a Three-Year-Old Girl

**DOI:** 10.7759/cureus.21683

**Published:** 2022-01-28

**Authors:** Jakob M Domm, Kirstin Weerdenburg, Renée Kinden, Jason G Emsley

**Affiliations:** 1 Emergency Medicine, Dalhousie University, Halifax, CAN; 2 Emergency Medicine, IWK Health, Halifax, CAN

**Keywords:** pediatric, refractory ventricular tachycardia, tachyarrhythmias, pediatric cardiac arrhythmias, ventricular tachycardia (vt), wide-complex tachycardia

## Abstract

Wide-complex, monomorphic tachycardias represent a range of tachyarrhythmias. Such patients can present asymptomatically and hemodynamically stable, while others are in shock. The etiology of the rhythm can be difficult to determine in the emergency department, and although electrocardiogram findings may be helpful, a workup after stabilization may be necessary to determine the cause. Treatment is therefore dependent on hemodynamic status and follows a stepwise approach, as initial therapies may be ineffective. We present the case of a three-year-old girl with wide-complex tachycardia which was exceedingly refractory to preliminary treatments and required trials of multiple treatment approaches to achieve conversion to normal sinus rhythm.

## Introduction

Wide-complex tachycardia (WCT) requires rapid assessment and urgent or emergent treatment as it can lead to hemodynamic instability and potentially death. WCT may be monomorphic or polymorphic. Generally, monomorphic WCT represents either ventricular tachycardia (VT) or supraventricular tachycardia with aberrancy (SVTa), and effectively discerning VT from SVTa can be difficult [1]. There are electrocardiogram (ECG) algorithms to help make this distinction [2], yet VT is often misdiagnosed as SVTa [3]. If VT is determined to be the cause, there remain various etiologies of VT with distinct risks and specific treatments.

The severity of symptoms exhibited by patients with VT varies considerably [4,5]. In adults, VT may often lead to clinical deterioration and is frequently associated with cardiovascular disease, ischemia or structural heart diseases such as cardiomyopathy or fibrosis [6]. On the contrary, VT in children is most commonly idiopathic and benign, but can be related to cardiomyopathy, structural heart disease, channelopathies or electrolyte imbalances, all of which carry increased mortality rates [4]. Monomorphic WCT in a hemodynamically stable patient, regardless of the patient’s age, may respond to a trial of vagal maneuvers [1,7,8], but rapid IV administration of adenosine [9], with the possible addition of a beta-blocker or calcium channel blocker infusion [10], and/or amiodarone/procainamide loading [9], may also be necessary. We present a case of monomorphic WCT due to VT in a previously healthy three-year-old girl that was significantly refractory to initial treatments, which illustrates that not all monomorphic WCT is equally responsive to routine treatment and medication adverse events, such as shock, must be monitored.

## Case presentation

**Figure 1:**
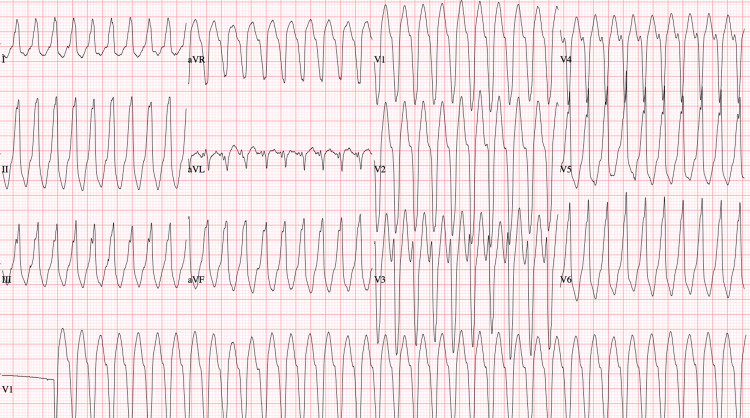
12-lead ECG obtained on presentation showing a wide complex, monomorphic tachycardia

A previously well three-year-old girl presented to a tertiary care pediatric emergency department with a two-day history of acute onset of intermittent abdominal pain and vomiting. She had no recent episodes of apnea, stridor, choking, syncope, sweating, increased work of breaking or weakness. There had been no changes in her activity level or appetite. She was otherwise healthy, and immunizations were up to date. Her birth was uncomplicated, she took no medications, and had no known allergies. There was no pertinent family history of cardiac disease or sudden death. This was her first presentation to the hospital since birth. On examination, she was distressed and crying but speaking clearly. She was pale but was not cyanotic. Her vital signs were as follows: heart rate 250 beats per minute (bpm), blood pressure 88/68 mmHg, temperature 36.5 °C, respiratory rate 30 breaths per minute and oxygen saturation 100% SpO2 on room air. Her cardiovascular, respiratory, and abdominal examinations were unremarkable. An ECG showed a monomorphic WCT (Figure 1). Electrolytes, complete blood cell counts and venous blood gas were normal (Table 1).

**Table 1:** Laboratory values Reference ranges are validated using the laboratory methods available at this children’s hospital and the unique patient population served. Therefore, they may not be applicable to other centers or different patient populations. pCO2: partial pressure of carbon dioxide; HCO3: bicarbonate

Variable	Reference range (index hospital and age group)	First presentation to ED	Second presentation to ED	Third presentation to ED
Hemoglobin (grams/liter)	102-127	133	128	130
Platelet count (x 10^9^/liter)	189-394	339	373	371
White-cell count (x 10^9^/liter)	4.86-13.18	9.2	8.78	9.62
Sodium (mmol/liter)	136-145	137.7	139	141
Potassium (mmol/liter)	3.4-5.0	4.5	4.0	4.7
Chloride (mmol/liter)	100-110	106.4	109	109
Magnesium (mmol/liter)	0.86-1.17	0.93	0.95	0.85
Calcium, ionized (mmol/liter)	1.20-1.38	1.31	1.28	1.26
Phosphorus (mmol/liter)	1.38-2.19	1.82	1.81	-
Venous blood gas				
pH	7.30-7.43	7.39	7.39	7.38
pCO2 (mmHg)	38-50	35.2	37.4	34.9
HCO3 (mmol/liter)	22-29	21.3	22.2	20
Lactate (mmol/liter)	0.5-2.2	-	1.0	-
Glucose, random (mmol/liter)	3.8-7.8	5.8	4.3	5.1

**Figure 2:**
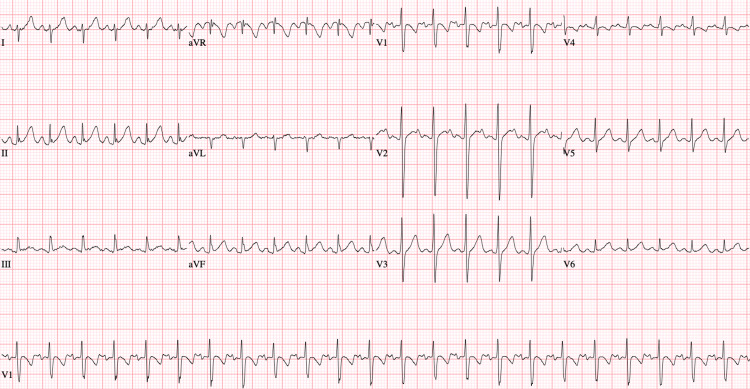
12-lead ECG obtained after conversion to normal sinus rhythm with procainamide load

The medical team initiated several vagal maneuvers (VMs), such as blowing into a straw, Trendelenburg position and ice on the forehead, which resulted in a transient reduction of the pulse to 180 bpm; however, it soon returned to 250 bpm. Next, adenosine was rapidly pushed through a peripheral IV at 0.1 mg/kg and then at 0.2 mg/kg, without effect. Cardiology was consulted and an esmolol infusion was started at 50 mcg/kg/min and then increased to 100 mcg/kg/min. However, this beta-blocker infusion did not achieve rate control and was discontinued after its use led to a transient episode of hypotension (70/49 mmHg). Ultimately, a procainamide load resulted in conversion to normal sinus rhythm (NSR) at a rate of 110 bpm (Figure 2). While in hospital, a chest radiograph and echocardiogram were normal, with no signs of myocarditis, cardiomegaly or structural abnormalities. Cardiac magnetic resonance imaging (MRI) did not demonstrate fibrosis, myocardial edema or structural abnormalities. Cardiologist follow-up ultimately determined the etiology to be idiopathic VT because of the characteristic VT morphology and absence of underlying bundle branch block or accessory pathway.

She was started on flecainide and was asymptomatic and in normal sinus rhythm on discharge. However, three days later she returned to the emergency department after being picked up from daycare with abdominal pain and had the same WCT. VMs were unsuccessful again and the rhythm was terminated a few times with adenosine but resumed shortly thereafter. A loading dose of procainamide was ultimately required to return to sinus rhythm. One week later, she presented to the emergency department one more time with the same WCT, until ultimately being started on oral nadolol at 1 mg/kg/day and flecainide at 3.3 mg/kg/day. To date, she has had no subsequent return of symptoms, and an outpatient Holter monitor revealed no significant ectopy or arrhythmias.

## Discussion

Pediatric tachycardia is generally managed in an algorithmic manner. First, it is important to assess the patient’s hemodynamic status and ability to maintain adequate end-organ perfusion. If the patient is unstable, urgent cardioversion is indicated. If stable, the next steps are to determine whether it is a wide- or narrow-complex tachycardia, and whether there is a monomorphic or polymorphic QRS. Wide- and monomorphic-complex tachycardias are often due to VT, but may also be caused by hyperkalemia, toxins [11] or underlying SVTa [1]. Differentiating VT and SVTa can be challenging. In the absence of electrolyte abnormalities or drug/poison exposures, the presence of capture beats, fusion beats and atrioventricular dissociation on ECG with a monomorphic WCT are more suggestive of VT rather than SVTa [1] and there are ECG algorithms to help make this distinction [2]. Furthermore, in adult populations, adenosine may be both therapeutic and diagnostic at differentiating VT from SVTa, as one study showed that 90% of SVTa was terminated by adenosine, whereas only 2% of VT was terminated by adenosine [12]. However, the common types and underlying causes of VT in the pediatric population markedly differ from those observed in adults, and have often been shown to be significantly more responsive to adenosine administration [8,13,14].

The most common type of VT in children is idiopathic VT (IVT) [4,15], which occurs in structurally normal hearts without a channelopathy or metabolic imbalance [10]. IVT is less likely to lead to hemodynamic instability than other types of VT and in one study was shown to have zero mortality or heart transplant rate, compared to combined heart transplant or mortality rates of 70% with cardiomyopathy, 32% with structural heart disease and 14% with channelopathy [4]. Further, IVT infrequently requires treatment, and any treatment utilized is generally for symptomatic management [4]. However, in a 25-year retrospective study, 11% of pediatric patients with IVT presented in shock and required urgent treatment [4]. Although more rare, VT secondary to cardiomyopathy, structural heart disease, channelopathies, or electrolyte imbalance requires prompt recognition and treatment as they have greater morbidity and mortality [4].

In the pediatric population, specifically, treatment of monomorphic WCT consists of synchronized cardioversion if unstable [9] and initial trial of VMs if stable, followed by medical management [1,8]. VMs such as carotid sinus massage, putting an ice-cold towel on the face, or the Valsalva maneuver ostensibly increase parasympathetic tone, hypothesized to cause acetylcholine release via the vagus nerve, reducing beta-adrenergic and cyclic adenosine monophosphate stimulation of the ventricular myocardium, thereby terminating sympathetic-triggered arrhythmias, such as SVT or certain types of VT [1,7,16]. In the case we present here, VMs were attempted with transient efficacy but were not successful at terminating the tachyarrhythmia. A similar phenomenon was observed elsewhere, when ice to the face, Valsalva maneuvers and retching were only able to temporarily decrease the heart rate in a patient with monomorphic VT, but were unable to terminate the rhythm until a significant coughing spell developed [7]. This may be because the coughing spell ultimately achieved the intrathoracic pressure required to reduce preload significantly enough to cause a vagal response upon breathing normally and a rush of blood into the heart. Interestingly, it has been suggested that VMs can terminate VT at a rate proportional to the intrathoracic pressure achieved by the patient [1,8].

If VMs are unsuccessful at terminating the monomorphic WCT, one should move forward with pharmacotherapy, typically with adenosine, which demonstrates effectiveness at terminating SVT and certain types of VT more commonly seen in pediatric patients [12-14]. Adenosine is proposed to work through decreasing beta-adrenergic and cyclic adenosine monophosphate stimulation of the ventricular myocardium, similar to VMs [17]. It is critical that it be administered quickly intravenously, as the drug must reach the myocardium, but has an extremely short half-life [7]. Generally, beta-blocker infusion [10] is the next line of medical therapy, followed by amiodarone/procainamide loading if required [9,18]. In this case, esmolol infusion was initiated but resulted in an episode of hypotension, so it was discontinued. This serves as an important reminder to consider the risks associated with classes of medications and monitor for adverse events accordingly.

The identification of monomorphic WCT on ECG requires further evaluation to differentiate amongst the various etiologies. In regards to VT etiologies, Channelopathies are generally inherited, present in 12% of pediatric VTs [4] and require thorough family history, exercise testing and/or genetic testing to diagnose. An echocardiogram can assess for both cardiomyopathy, occurring in 17% of pediatric VTs [4], as well as structural heart disease, which comprises 16% of pediatric VTs [4]. MRI may also be used to better qualify structural heart disease and/or assess for fibrofatty infiltrates [10]. Regardless, pediatric patients with VT require close follow-up as their rhythm may not be benign. Even if they ultimately are determined to have IVT, children with IVT commonly have a recurrence of symptoms and arrhythmia following the first episode [4], as was observed in the present case.

## Conclusions

Monomorphic WCT may represent VT, SVT with aberrancy, electrolyte imbalance or toxin exposure and could be benign or fatal depending on the cause. In pediatric patients presenting to the emergency department with the first episode of monomorphic WCT, the etiology and type of tachyarrhythmia is difficult to determine, and treatment should be pursued with a stepwise approach, initially based on whether the patient is unstable and requiring electrical conversion. If the patient is stable, a trial of VMs or adenosine should be pursued. However, cardiologists may need to be involved as monomorphic WCT can be refractory to initial treatments, as was shown in the current case, and further treatments and close monitoring may be necessary. Further evaluation after stabilization to determine the etiology of WCT is often necessary.
